# Remarks on the Practical Utility of Galvanism, or Metallic Electricity, with a View to Ascertain Its Effects on Muscular and Nervous Irritability

**Published:** 1799-03

**Authors:** 


### *Remarks on the practical Utility of Galvanism, or Metallic Electricity, with a View to ascertain its Effects on muscular and nervous irritability.*

THE experiments of GALVANI, on the irritability of the muscular and nervous fibre, by the well-known application of metallic stimuli, have long excited the curiosity of medical practitioners, and induced many of them to address the query—*Cui bono?* At length M. VON HUMBOLDT has published his "*Experiments on the stimulated muscular and nervous Fibre, with Conjectures on the chemical Process of Life in the Animal and Vegetable Kingdoms,*" (in German) Vol. I. 8vo. pp. 495, with eight plates. The very ingenious author of this work is considered in Germany as the *founder of a rational system of* of ANIMAL CHEMISTRY. The nature and tendency of his unremitted inquiries will be more clearly understood from the following characteristic passage extracted from the preface:—"For several years," says he, "I have been assiduously employed in comparing some phœnomena of animal matter, with the laws of inanimate nature. During these researches, I have succeeded in experiments, which promise to conduct us somewhat farther in discovering the chemical process of life. I have found that the separated organ of an animal, being provided with irritable and sensible fibres, may in a few seconds be raised from a state of the most complete insensibility, (inexcitability) to the highest degree of susceptibility of a stimulus, and *vice versa.* This alternate change of increased and diminished vital power may be produced in a nerve, four or five times, by the same act of volition, with which the hand of the artist extends or relaxes the strings of an instrument. I have stimulated the organs of animals with oxygenated muriatic acid, alkalies, nitrous acid, the calx of arsenic, opium, and alcohol, for some hours successively, and yet they emerged from these struggles with the the contending elements, almoſt without any material change of their nature. I have obſerved, that animal bodies poſſeſs the power of operating from a diſtance, and have endeavoured to exhibit to the ſenſes this circle of action, which decreaſes together with the power of life. I think I can demonſtrate, that the irritability of matter does not depend upon the quantity of *oxygen* alone, as is conjectured by modern phyſiologiſts, and as my own experiments upon plants appear to inſinuate; but that *azote* and *hydrogen* have a more important ſhare in this power; and that every thing herein depends upon the *common operation* and the *antagoniſm of a plurality of ſubſtances.*"

We ſhall not attempt here to abridge this work, or to give an abſtract of the numerous experiments inſtituted by the author, on animal and vegetable ſubſtances, under the moſt diverſified circumſtances and relations, as the taſk would be too complicated and laborious. The reader will be better gratified by learning the general reſult of Mr. Humboldt's inquiries into the *practical utility* of *Galvaniſm;* for the metallic ſtimulus invented by Galvani has been lately exhibited by another German writer, Mr. CREVE, as the moſt certain criterion for diſtinguiſhing *aſphyxia* from actual death. Humboldt is of opinion that this ſtimulus is not an infallible mode of aſcertaining death in caſes of aſphyxia; becauſe, 1. the electric fluid ſtill evinces ſymptoms of the ſuſceptibility of ſtimulus in a nerve, which can no longer be ſenſibly affected by that of Galvani; 2. becauſe the experiment can be made only on *ſome* parts of the body, and the want of excitability in theſe, does not infer the ſame defect in the *whole* nervous ſyſtem; 3. becauſe we have inſtances in which the metallic ſtimulus has proved ineffectual in thoſe organs which ſhortly before, and even after its application, could be voluntarily moved; and, 4. as it is a matter evidently doubtful, that parts which for a time appear to be deprived of all irritability, can in the ſequel again recover it.

